# Early Detection of Subsurface Fatigue Cracks in Rolling Element Bearings by the Knowledge-Based Analysis of Acoustic Emission

**DOI:** 10.3390/s22145187

**Published:** 2022-07-11

**Authors:** Einar Løvli Hidle, Rune Harald Hestmo, Ove Sagen Adsen, Hans Lange, Alexei Vinogradov

**Affiliations:** 1 Department of Mechanical and Industrial Engineering, Norwegian University of Science and Technology—NTNU, 7491 Trondheim, Norway; einarhidle@outlook.com; 2 Water Linked AS, 7041 Trondheim, Norway; 3 Kongsberg Maritime AS, 7053 Trondheim, Norway; rune.harald.hestmo@km.kongsberg.com (R.H.H.); ove.adsen@km.kongsberg.com (O.S.A.); 4 Materials and Nanotechnology, SINTEF Industry, 7465 Trondheim, Norway; hans.i.lange@sintef.no

**Keywords:** fault diagnostics, acoustic emission, data processing, rolling contact fatigue, subsurface crack

## Abstract

Aiming at early detection of subsurface cracks induced by contact fatigue in rotating machinery, the knowledge-based data analysis algorithm is proposed for health condition monitoring through the analysis of acoustic emission (AE) time series. A robust fault detector is proposed, and its effectiveness was demonstrated for the long-term durability test of a roller made of case-hardened steel. The reliability of subsurface crack detection was proven using independent ultrasonic inspections carried out periodically during the test. Subsurface cracks as small as 0.5 mm were identified, and their steady growth was tracked by the proposed AE technique. Challenges and perspectives of the proposed methodology are unveiled and discussed.

## 1. Introduction

### 1.1. Background and Problem Statement

Rolling element bearings and gears are extensively used in heavy-duty applications across almost all global industries. Rolling element bearings and gears are susceptible to multiple forms of damage, including corrosion, denting, electrical erosion, fracture, and spalling [1]. Failures in these critical components can often result in catastrophic accidents with potentially massive social, economic, and environmental ripple effects. Therefore, proactive strategies based on real-time condition monitoring and early detection of incipient damage are being developed to prevent non-scheduled shutdowns, catastrophic failures, and production losses [2,3,4,5].

Under normal operation conditions, the elements of bearings and gears, such as rollers, raceways, and gear tooth flanges, are exposed to multiaxial and non-proportional, high cyclic loadings. As a result of design complexity, multiple intrinsic and extrinsic factors affect the service life of rotating components. These factors include (but are not limited to) material and lubricant properties, load, the geometry of the assembly, rotation speed, etc. Thus, even when operating normally, bearings and gears will eventually fail as an inevitable result of contact fatigue phenomenon [6]. Contact fatigue damage often initiates as a subsurface crack at local structural inhomogeneities and shear stress risers, which are introduced by a contact pressure at the surface. The crack then grows and propagates towards the surface, giving rise to spalling and crumbling in bearings and gear components [7,8], and ultimately causing failure of the entire structure [6]. The failure starts to evolve unpredictably after some variable time of service, as embryonic subsurface cracks are extremely challenging to reveal during service. When it reaches a critical size at the surface of the component, the fault is detectable by a wealth of vibration and acoustic emission (AE) techniques [9,10,11,12,13,14,15,16,17,18,19,20], or a combination of both. These techniques are powered by contemporary signal processing algorithms [21,22] and/or other non-destructive testing methods, such as the ultrasound technique [23], Barkhausen noise analysis [24], shock-pulse methods [25], infrared thermography [26,27,28], oil monitoring [29], etc., or a combination of these methods [30,31]. However, it is fair to say that vibration analysis currently prevails in the field of monitoring rotating machinery, including the rolling bearing fault diagnosis [11]. Despite its obvious advantages associated with analysis based on fundamental bearing frequencies [32], this method is limited to systems operating at relatively high speeds. However, many heavy-duty bearings and gearboxes operate at low speeds where surface vibrations are hardly detectable. In addition, vibration-based techniques are sensitive almost exclusively to surface defects. That is, the insipient subsurface cracks induced by rolling contact fatigue are unlikely to be detected by vibration methods until the cracks reach the surface of the rotating machine component and cause a surface defect. This is because most of the remarkable changes in vibration signals occur mainly due to modification of the contact surface geometry [11,33]. However, at the moment when the damage reaches the surface and becomes detectable, minimal time (if any) is left for maintenance and repair. The decisive benefit of AE compared to vibration methods (as well as to other non-destructive techniques used for condition monitoring of rotating machinery) is its unique capacity to follow the initiation and propagation of fine dynamic defects regardless of their place of origin—surface or subsurface [34]. The AE technique is capable of detecting transient elastic surface waves caused by released strain energy during elementary processes of plastic deformation in metals [35], crack initiation and growth, and friction and wear sources [36,37,38].

T. Yoshioka [39] first indicated the possibility of detecting acoustic emissions stemming from subsurface fatigue cracks induced 50–200 μm below the surface of the raceway in running ball bearings (see also [40]). The location technique capable of finding the stationary position of AE sources on the raceway was used to this end. Since then, despite significant efforts invested into this topic by the research community, detecting incipient subsurface fatigue cracks as the earliest harbingers of imminent failure remains acute and challenging. In 2005, Price et al. [41] presented results showing the advantages of the continuous AE recording for fault detection in heavily loaded specimens during four-ball testing. Before pitting occurred at the free surface, a distinct shift in AE energy from the peak (at approximately 115 kHz) to the peak at a lower frequency (of 50 kHz) was observed. The post-mortem autopsy of the balls showed subsurface crack networks leading to the pits, thus indicating a possibility of identifying the subsurface damage. Using the time-frequency analysis of AE in the four-ball tribology testing scheme, Lees et al. [42,43] made a similar conclusion. Recently, using seeded subsurface cracks introduced to a raceway by means of compressing its outer surface with a rolling element, Fuentes et al. [44] took the next step forward in developing monitoring methods capable of detecting subsurface damage in rotating machinery. These authors demonstrated this possibility in an elegant way by employing the probabilistic Gaussian mixture models applied to hit-based AE features, including hit summary statistics, auto-regressive coefficients of the individual AE hit time histories, and the envelope spectra of raw AE signals. The proposed approach, however, has not been verified for practical cases when the subsurface damage initiates and evolves naturally as a consequence of the rolling contact fatigue (RCF) in the initially undamaged bearings. Subsurface cracking is readily observed as a key feature and prevailing mechanism in the very high cycle fatigue of structural materials [45,46], including bearing steels [27,28]. Recently, it was shown [47] that the contemporary AE technique powered by the temporal-frequency short-time Fourier analysis is capable of detecting subsurface damage initiated during the laboratory ultrasonic fatigue testing in the gigacycle regime. Nevertheless, none of the methods proposed in the above-cited works can be regarded as robust enough to become a widely deployed and effective tool for condition monitoring systems capable of reliable subsurface crack detection in noisy industrial settings. A new flavour and additional dimension to routine data processing and condition monitoring is added by burgeoning machine learning approaches [5,48,49,50,51]. Despite the rapid progress in the field, the typical black-boxed properties of inference mechanisms involved in machine learning models make outcomes hard to verify, thus preventing the concept from reaching a level of acceptance and credibility that assists in its adoption or adaptation in the industry outside of some niche applications. Thus, subsurface crack detection in rolling element bearings and gears, using the acoustic emission time series, remains an important issue in the condition-monitoring world.

The objective of the present work is to develop a robust methodology for AE time series analysis aiming at condition monitoring and early diagnosis of faults induced in rolling element bearings during contact fatigue loading. The prime novelty lies in a combination of knowledge-driven feature extraction based on windowed pulse integration and the original verifiable decision-making protocol. Using the developed signal processing toolbox enabled us to detect and prove the appearance of subsurface contact fatigue cracks through AE waveform analysis with high confidence for the first time, to the best of our knowledge.

### 1.2. Methodology

The condition-based monitoring and industrial predictive maintenance strategy (as opposed to run-to-failure maintenance), in general, involves four main stages: (i) data acquisition, (ii) data processing aiming at constructing the so-called health indicators, (iii) determination of the decision threshold, and (iv) decision making through the detection of an anomaly in the signal behaviour [52,53]. Data acquisition is the process of collecting sampled sensor data such as AE, vibration and ultrasound data, temperature, ambient moisture, etc. At the data processing stage, relevant features associated with health indicators are extracted from raw data and classified to separate the effects of damage from those of normal operation and outliers created by a variety of sporadic external or internal sources of noise (friction and wear, roller impact, misalignments, splashing oil, electric interferences, etc.). The categorised extracted features then serve as inputs for the decision-making stage according to the chosen decision threshold.

The problem of extracting health indicators from condition monitoring signals has long been recognised as a central issue that currently remains acute despite significant efforts to address it with or without aid from artificial intelligence [54]. Hundreds of features and related statistical techniques were proposed and tested in a traditional vibration-based fault diagnostics domain [52,55,56,57,58,59,60,61]. A similar picture is seen in the evolution of a family of rapidly evolving acoustic emission methods [62,63,64,65,66,67,68,69]. Fusion of information is often used to mitigate the uncertainty and shortcomings of individual techniques by combining the incomplete and imperfect pieces of mutually complementary sensor data, thereby raising confidence in decision-making.

The acoustic emission from rotating machinery can be described as a random waveform with a continuously changing noise floor. Rolling contact fatigue-induced cracks manifest themselves in the waveform as short bursts of energy, or low-amplitude pulses, which can be completely hidden in the severe background noise. The general problem of finding these pulses [70], which was reviewed and addressed with an account of specifics of AE signals in [71] (see also [72,73]), reduces problems dealing with periodically re-appearing signals to a narrower class of detection. A recently emerged branch of signal processing called cyclostationarity [74,75,76] represents an influential statistical theory developed for analysing signals that contain a cyclic pattern of statistical features. It is, therefore, extremely useful when dealing with waveforms that have hidden periodicities. In a narrower sense, the problem is similar to that commonly faced in the radar target detection problem [77,78]. Inspired by radar target detection theory and methods, the approach proposed in the present work employs a robust detector capable of independent detection of multiple RCF-induced subsurface cracks occurring in an operating rotating machine. The outputs from the detector are easily verifiable; therefore, decision-making is arguable with high confidence.

In the following discussion, we endeavour to demonstrate that incipient subsurface fatigue cracks can be detected via the analysis of acoustic emission time series obtained during a roller element durability test.

The rest of the paper is organised as follows. Section 2 presents the experimental setup and testing schedule, AE recording system. Mathematical description of the proposed detector is given in Section 3. Section 4 demonstrates the evolution of damage assessed by the AE technique during the durability test, detector decisions, and verifications. In Section 4, the main findings are interpreted and discussed. The proposed detector’s performance is evaluated and compared to the behaviour of the most commonly extracted AE feature—root mean square (rms) voltage. Finally, conclusions are drawn in Section 5.

## 2. Experimental

### 2.1. Testing Rig, Specimen and Loading Conditions

**Figure 1:**
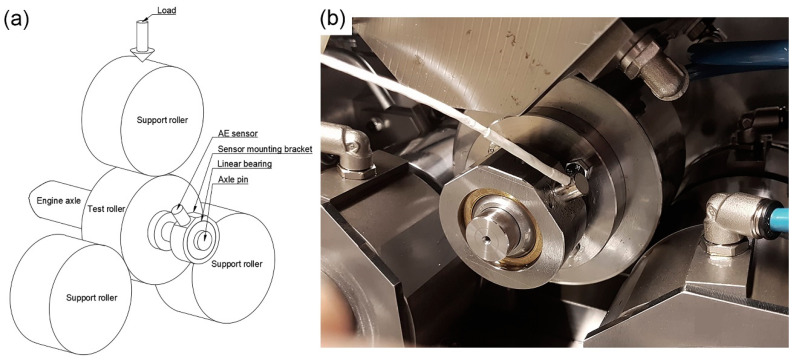
(**a**) Schematics and (**b**) the photographic image of the part of the test rig showing the test roller, the support rollers, and the AE sensor mounting.

To monitor the rolling contact fatigue (RCF) phenomenon occurring in a loaded rolling machine element, such as bearing elements or gear tooth flanges, a run-to-failure test was carried out using an instrumented special-purpose rolling fatigue test rig designed by one of the co-authors (H.L.) at SINTEF Industry (Trondheim, Norway). The experimental setup is schematically illustrated in Figure 1. The test specimen (central roller, made of case-hardened gear steel) is supported by three support rollers (60 HRC through-hardened tool steel), and each support roller is supported by two needle bearings. The diameter of the rollers is ø115 mm. The case-hardening depth of the test roller is 1.0 mm, which is equivalent to the gear module of a typical large spiral bevel gear. The contact surface of the support rollers has a 3750 mm radius curvature to avoid any edge effects. The surface roughness of the contact surfaces is RZ ≤ 0.4 µm. To control the contact stress, a load cell is mounted above the upper support roller. An axle pin is bolted to the test roller on the opposite side from the shaft coupler (Figure 1b), and the broadband WD sensor (MISTRAS, Princeton, NJ, USA) is mounted on a linear bearing mounted to the axle pin. The AE sensor is attached to the surface of the bearing house with a constant force created by a spring, mounted inside the sensor holder, which is bolted to the sensor mounting bracket by two screws. A high-temperature grease GLEITMO 591 (Fuchs Lubritech GmbH, Kaiserslautern, Germany) is used as a contact medium between the sensor and sensor mount. The linear bearing, needle bearings, and contact points between the test and support rollers are all lubricated with SP100 gear oil supplied from the rig’s oil management system. The oil is continuously filtered with a 4 µm oil filter. The electrical signal from the AE sensor output was amplified by 40 dB in the frequency band 20–1200 kHz by the 2/4/6 low-noise preamplifier (MISTRAS, Princeton, NJ, USA) and transferred to the high-speed data acquisition system HSIO-100-A developed by Kongsberg Maritime. The HSIO-100-A unit is based on the high-resolution 24-bit high accuracy analogue-to-digital converter operating in a continuous streaming mode at 2 Msamples/s frequency.

A point on the perimeter of the test roller passes three contact points at the support rollers per rotation of the test roller due to the triangular symmetry of the loading system. That is, three fatigue cycles occur per axle rotation. The corresponding “test frequency” is, therefore, defined as *f*_*text*_ = 3*f*_*r*_, with *f*_*r*_ = $F_{A}$/60, where $F_{A}$ is the axle rotation frequency defined in rpm.

### 2.2. AE Acquisition System and Test Schedule

Before any significant loading was applied to the test roller during testing, a warmup stage was performed. Monitoring the oil temperature, the test rig was operated using stepwise uploading until the oil temperature stabilised at 50 °C. The initial axle rotation frequency was then set to $F_{A}=364\;$rpm, and the initial load was set to 67 kN, which corresponded to 1800 MPa contact stress on the rollers. The test was interrupted periodically for ultrasonic inspections using the OMNISCAN SX (Olympus, Tokyo, Japan) phase array ultrasonic tester (PAUT). As PAUT inspections revealed no faults after initial cycling, the load was gradually increased in a stepwise manner up to 91 kN (2000 MPa contact stress) after 2.7 × 10^7^ cumulative fatigue cycles. Sudden excessive vibrations were detected in the axle flex coupling during the first test period at 2000 MPa (91 kN) contact stress. The rotation frequency, $F_{A}$, was thereafter reduced to 256 rpm until the end of the duration test, and the contact stress was kept constant at 2000 MPa. The first subsurface crack of 0.5 mm length was detected by the ultrasonic technique after 2.8 × 10^7^ fatigue cycles (i.e., 1.1 × 10^6^ fatigue cycles at 2000 MPa contact stress). The crack then slowly expanded to approximately 5 mm length and 10 mm width before the test was terminated after the accumulation of 6.7 × 10^7^ fatigue cycles (in the order 1.6 × 10^7^ at 1800 MPa, 1.4 × 10^7^ at 1900 MPa, and 3.6 × 10^7^ at 2000 MPa). The test roller was then sectioned for metallographic inspections and verification of both PAUT and AE results.

AE waveforms were continuously recorded at 2 MHz sampling frequency for 2 s per record. At the beginning of the test, AE streams were collected by timer every 60 min. After confirmation of the first subsurface crack by the PAUT inspection, the time interval between the successive AE acquisitions was reduced to 20 min.

## 3. Proposed Detector

The detector described in this paper was introduced in the master’s thesis of one of the present authors—E.L. Hidle [79]. In what follows, we provide a brief, yet self-consistent description of the signal processing methodology used. For the complete mathematical description, readers are encouraged to review the reference [79].

**Figure 2:**
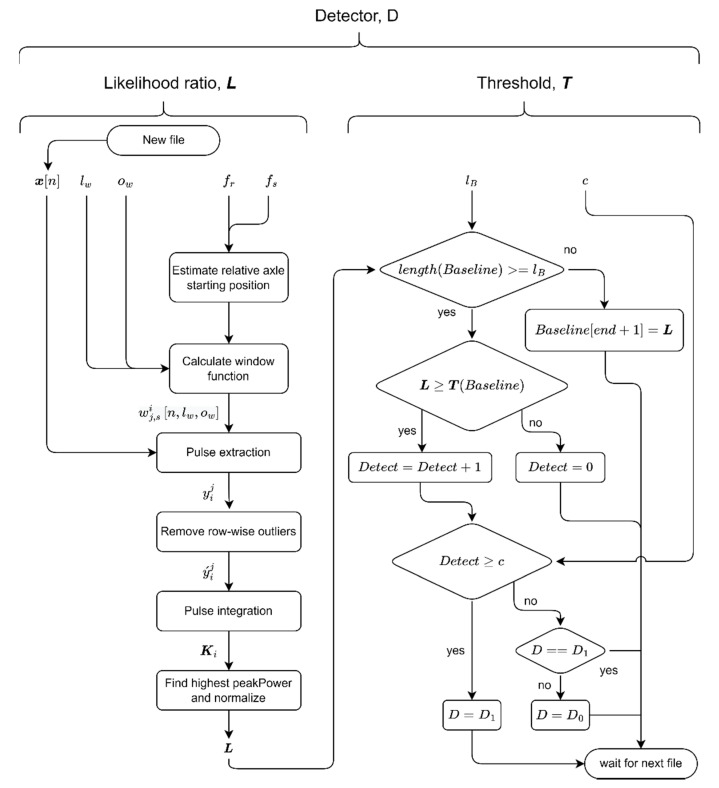
The flowchart illustrating the essential steps of the detection process (all variables in functions are defined in the text below).

A flowchart representing the workflow followed is illustrated in Figure 2. Data processing involves two primary stages—constructing the powerful health indicator (left-hand side) and decision-making (right-hand side). Data processing details are unfolded in subsequent sections.

### 3.1. Hypothesis Testing

Aiming at developing an automated detection process, the detector is built around a well-established decision theory. Decision making is a branch of statistics that describes the process of mapping noise-contaminated input data to a decision about the state of a system. The detector considers two possible alternative hypotheses, $H_{0}$ and $H_{1}$. In application to rotating machines, we deal with a binary hypothesis testing problem, where the null hypothesis, $H_{0}$, describes normal machine operation, and $H_{1}$ refers to the alternative situation where a fault (surface spall, subsurface crack, etc.) is present in the rotating machine. Given these hypotheses and the input data, x, the detector makes a decision, $D_{i}$, i∈0,1, prioritising one of two hypotheses [70]. The hypothesis is often tested by a likelihood ratio method [80]. The decision-making rule is based on the popular Neyman–Pearson (NP) criterion, which merely represents the condition of a minimum of the error probability of one kind at a given error probability of the other kind. In this criterion, the probability of a false alarm, $p_{fa}=p\left(D_{1}|H_{0}\right)$, is set as high as the operator decides to tolerate, thus maximising the probability of detection, $p_{D}=p\left(D_{1}|H_{1}\right)$. Effectively, the detector decides $D_{1}$ if the likelihood ratio, L(x), is equal or greater than a certain threshold, T [78], i.e.,

(1) $$L\left(x\right)=\frac{p\left(x|H_{1}\right)}{p\left(x|H_{0}\right)}\geq T$$

and the decision, $D_{0}$, is made otherwise (compare Figure 2). Thus, the likelihood ratio, L(x), which will be specifically defined in the following sections, serves as a health indicator in the present work.

### 3.2. Pulse Integration Method

The core procedure for building a powerful health indicator centres on the pulse integration method, which is implemented in several successive steps highlighted in Figure 2. In systems where multiple pulses form a train with a hidden periodicity, this method can be utilised to magnify the significance of pulses that emerge at specific time intervals. In other words, when the period from one pulse to the next one is known (or predictable) and constant, several pulses can be *integrated* within a certain time interval to achieve improved detectability. This procedure can be efficient even for time series with a very low signal-to-noise ratio (SNR), enabling the detection of low amplitude transients buried in heavy noise [77]. For the NP-based detector, the pulse integrating detector is reduced to the square-law detector, where $D_{1}$ is decided if the likelihood ratio is equal to, or greater than, T. Equation (2) thus takes the form

(2) $$L\left(y\left[n\right]\right)=\sum_{k=1}^{N}y^{2}\left[n,k\right]\geq T$$

where y[n] is the filtered signal vector x[n], and k is the sequential number of the pulse.

The advantage of using the pulse integration technique is understood by noting that integration is a process that reduces variance. If N independent noise samples are averaged, the standard deviation to mean ratio of L is reduced by $\sqrt{N}$ relative to the variation in y. Thus, the SNR is improved by reducing the noise rather than enhancing the signal itself [78].

### 3.3. Detection Stage

The detection algorithm represented by the left branch of the flowchart in Figure 2 is briefly described in this section.

#### 3.3.1. Window Function

**Figure 3:**
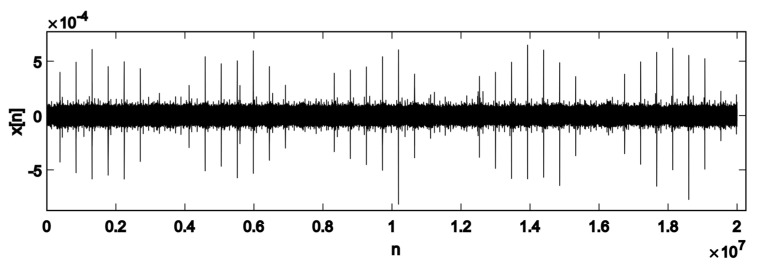
An example of the x[n] vector, sampled at $f_{s}=2\;\mathrm{MHz}$ for a total length of $l_{signal}=10f_{s}$ readings. Pulses repeating themselves at the fault frequency $f_{fault}=1/rev$ are visible as peaks in x[n].

The typical AE record, which is fed to the initial analysis block represented in Figure 2, is shown in Figure 3. Let the realisation x[n] illustrated in this figure be a sampled AE waveform of length $l_{signal}$, where $n=\left\{1,2,3,\ldots ,l_{signal}\right\}\;$is the number of readings. The record contains AE pulses repeating at various fault frequencies such as *f_fault_* = 3/*rev* and *f*_*fault*_ = 1/*rev*. The first step is to find the locations in the *n*-space where the pulses originated from the same faults (surface or subsurface). This is accomplished using a sliding window function with a rectangular shape. The window function can be interpreted as a matrix where each row corresponds to one window (and one distinct axle position), and the entries in each row correspond to sub-windows of each window (see Table 1).

From one row to the next, the sub-windows are shifted in the n-space with a sample distance equal to the difference between the window length, $l_{w}$, and the window overlap, $o_{w}$. The number of sub-windows is a product of the number of axial revolutions recorded in each file and the number of times a subsurface crack is expected to excite an AE pulse per axle revolution ($f_{fault}$). If $f_{fault}$ is an integer, each sub-window will capture the recorded signal at the same angular position of the axle. The recorded signal captured by each sub-window in each row is used for pulse integration.

#### 3.3.2. Pulse Extraction

**Figure 4:**
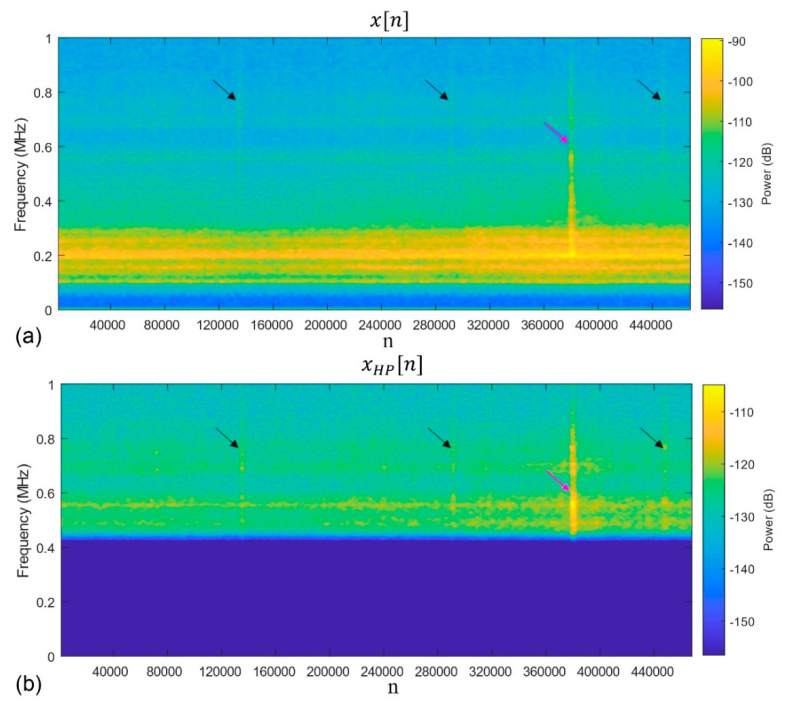
The spectrogram for (**a**) x[n] and (**b**) $x_{HP}\left[n\right]$, illustrating the noise filtering procedure (compare (**a**,**b**) with and without the low-frequency components in the spectrograms, respectively). The black arrows indicate the AE pulses repeating at $f_{fault}=3/rev$, and the pink arrows indicate the AE pulses repeating at $f_{fault}=1/rev$.

The sampled input signal vector, x, is zero-mean. The envelope detector is chosen to extract the AE pulses. This detector comprises a band-pass filter, a rectifier, and a low-pass filter. The acquired signal is high-pass filtered digitally, with a cut-off frequency at 500 kHz to remove the strong low-frequency components of the noise generated by the testing rig (the cut-off frequency is chosen from the spectrogram and can vary flexibly). Figure 4 illustrates the filtering procedure applied to a Fourier spectrogram of the signal fragment corresponding to one axle revolution.

The signal is further rectified by squaring the high-pass filtered components, $x_{HP}$. The final step is to sum all $x_{HP}^{2}$ components inside the sub-window, which is equivalent in a sense to low-pass filtering. Thus, the pulse extraction procedure reads as

(3) $$y_{i}^{j}=\sum_{n=1}^{l_{signal}}w_{i}^{j}\left[n\right]x_{HP}^{2}\left[n\right]$$

where $y_{j}^{i}$ refers to the extracted pulses, $w_{j}^{i}\left[n\right]$ is the window function, i refers to the window’s number, and j refers to the sub-window’s number.

#### 3.3.3. Outlier Removal and Pulse Integration

This is the final procedure before pulse integration according to the flowchart shown in Figure 2. When pulses from multiple subsurface crack sources occur simultaneously in the AE waveform, measures must be thoroughly exercised to ensure that the AE pulses sought are the pulses that trigger the detector. We expect pulses originating from the same distinct source to be relatively uniform in their magnitudes. If the outliers are captured in the same window with the signal, the amplitudes of outliers in the time series are replaced by the mean amplitudes of the pulses, which are not considered outliers. This ensures that the detector captures the specific temporal behaviour of pulses independently and separately from other possible behaviours occurring simultaneously in the rotating machine. After the outliers are removed, the pulses, ${\overset{´}{y}}_{i}^{j}$, are integrated as

(4) $$K_{i}=\sum_{j=0}^{N}{\left({\overset{´}{y}}_{i}^{j}\right)}^{2}$$

where $K_{i}$ is referred to as *K*-spectrum, and N is the number of sub-windows.

**Figure 5:**
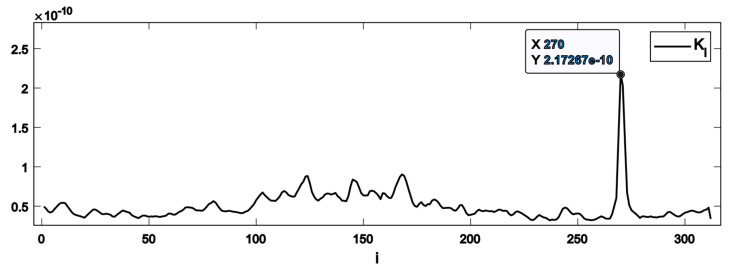
An example of the *K*-spectrum. Here the index, i**,** denotes the sequential number of the windows. It is only related to a specific axle angular position if the sought behaviour excites an AE pulse once per revolution of the axle ($f_{fault}=1/rev$ ).

Figure 5 shows a typical example of the *K*-spectrum with the high prominence sharp peak at i=270, which is indicative of the appearance of a fault (a subsurface crack in our case).

#### 3.3.4. Health Indicator, Likelihood Ratio (*L*), Threshold (*T*), and Decision (*D*)

The sought AE pulses originating from subsurface cracks give rise to a single narrow peak in the *K*-spectrum. To differentiate these peaks from other sporadic peaks, the property called peakPower is defined as the peak prominence divided by the peak width (peak prominence should not be confused with peak height). The prominence of a peak measures how significantly a peak differs from the surrounding baseline of the signal; peak prominence is defined as the vertical distance between the peak and its lowest contour line. Only *K*-spectrum peaks with the highest *peakPower* are considered as possible candidates for fault indicators. Furthermore, if the sampled signal, x[n], has a high noise content, the area under the curve in the *K*-spectrum will be larger in general, and the probability of the false alarm, $p_{fa}$, will increase. The likelihood ratio, L, which serves as the health indicator in the present work (Figure 2) thus takes the normalised form

(5) $$L=\frac{peakPower}{\int^{\;}K_{i}di}$$

The noise floor and general AE activity will differ substantially for different rotating machines. To define the threshold, T, the method chosen is to establish an upper bound based on the temporal sequence of L_s_. The idea is to record AE waveforms while the rotating machine is in its healthy state, establishing a baseline for the normal amount of AE activity for a particular machine. The baseline is defined as $BL=\left[L_{0},L_{1},\;\ldots ,\;L_{lb}\;\right]$, where *lb* is the chosen length of the baseline vector. Based on the created BL vector, the threshold, *T*, is defined by the following pseudocode (compare Figure 2):

T=3×mad(BL)+median(BL), where mad refers to the scaled median absolute deviation, which is defined for a random variable vector as

mad(BL)=s×median(|BL−median(BL)|) with the scaling factor $s=-\frac{1}{\sqrt{2}}erfcinv\left(\frac{3}{2}\right)$ and erfcinv is known as the inverse complementary error function [81].

Although the above measures were taken to prevent noise and unrelated AE activity from interfering with the likelihood ratio, L, sporadic AE activity may still cause undesired fault detections with L>T. To eliminate or account for these unexpected/unwanted detections, a confidence parameter, c, is introduced to the detection scheme to ensure that detections represent a stable breakpoint in the AE trend rather than random detections. That is, the positive decision $D_{1}$—*defect detected*—is made if the pre-set number of detections, c, are observed consecutively. Thus, the final expression for the detector takes the form

(6) $$D\left(L,T,c\right)=\{\begin{array}{cc} D_{1} & \left[L_{-c},L_{-c+1},\ldots ,L_{-1},L\right]\geq T\\ D_{0} & otherwise \end{array}$$

### 3.4. Pulse Integration Spectrum—A Novel Verification Procedure

**Figure 6:**
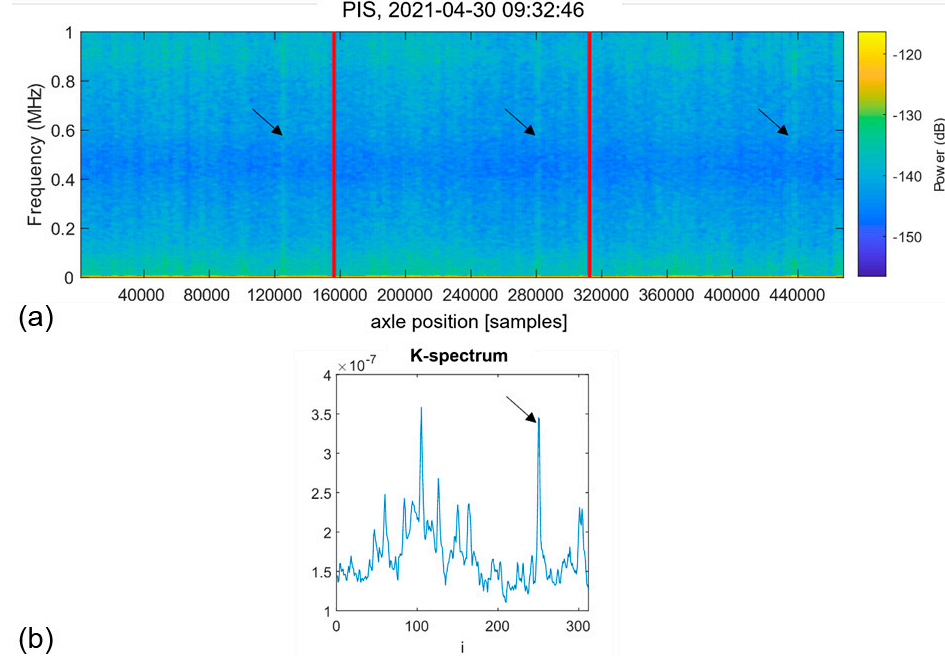
Example of the pulse integration spectrum corresponding to one axle revolution divided into three segments of equal length and marked by vertical red lines (**a**) and the *K*-spectrum corresponding to the middle segment (**b**) highlighting the presence of damage recurring in the waveform with the $f_{fault}=3/rev$ frequency. Characteristic spectral lines are indicated by black arrows.

Visual inspections of *K*-spectra provide a useful insight into the data structure that can be classified according to the presence of stable peaks (compare Figure 5), indicating the possibility of having a fatigue-induced fault in the testing piece. However, the *K*-spectrum-based detector alone does not allow verification that the positive decision originates from the sought AE behaviour corresponding to fatigue-induced faults. Therefore, a verification tool, the Pulse Integrated Spectrogram (PIS), is introduced here to address this challenge [79]. The process of constructing the Pulse Integrated Spectrogram is similar to the procedure implemented in the detector, albeit with some essential modifications. Instead of the window function, the sampled AE waveforms from complete axle revolutions are pulse-integrated. The output is then transformed to the time-frequency domain through the short-time Fourier decomposition, yielding a spectrogram of the pulse-integrated waveform. This representation will magnify the AE activity that occurs only at the same angular position of the axle during operation. The significance of AE activity occurring at random axle positions will be reduced. An example of the PIS revealing the characteristic hidden periodicity in the appearance of AE pulses is shown in Figure 6a, and the corresponding *K*-spectrum is represented in Figure 6b. Note that similar to Figure 5, the *K*-spectrum referring to the spatial coordinate denoted by the index, *i*, exhibits a sharp peak indicated by the black arrow. This suggests that there is a defect in the test piece. There are three clearly visible vertical lines (marked with black arrows) in the PIS, which are located at the same relative position with respect to the vertical red grid marking the initial axle position. This suggests that there is a defect in the test piece, which manifests itself at every interaction with the supporting rollers at $f_{fault}=3$. Thus, the hidden periodicity with the sought frequency is verified without doubt.

Figure 6 illustrates that the peak with the largest peak height in the *K*-spectrum does not necessarily correspond to the sought defect. Thus, the prominence, or *peakPower,* is used instead of the height as a criterion for inclusion or rejection from the analysis.

## 4. Results and Discussion

### 4.1. Application of the Proposed Detector to the Roller Bearing Test

**Figure 7:**
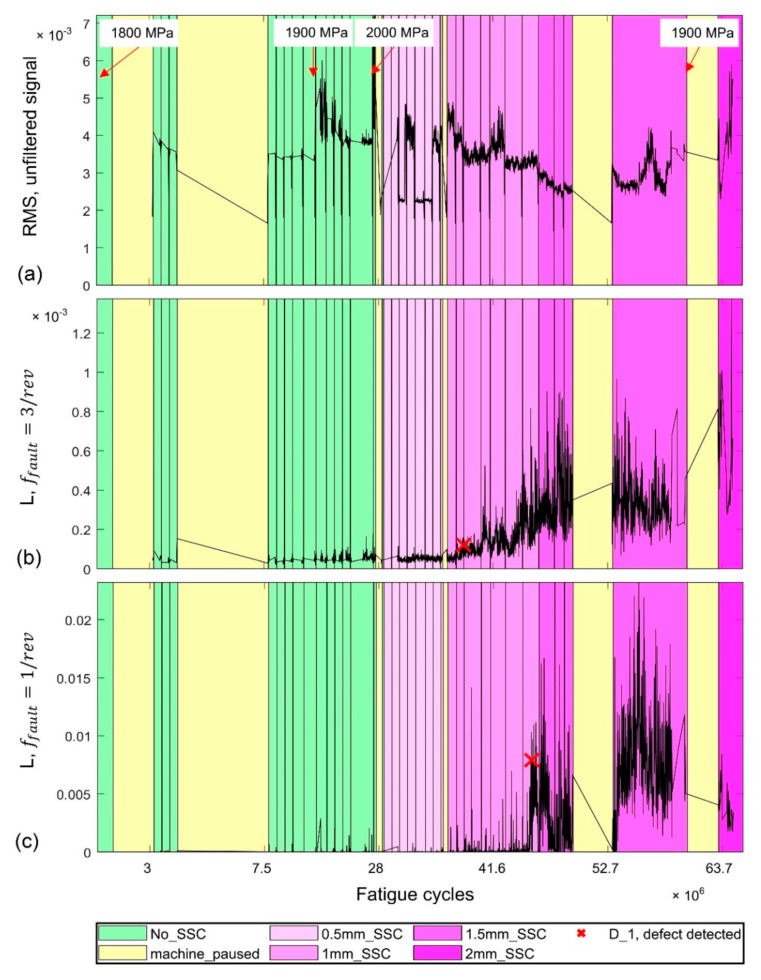
The AE behaviour during the durability test: (**a**) the conventional AE rms value as a function of the number of fatigue cycles; the likelihood ratio, *L*, corresponding to two fault frequencies (**b**) $f_{fault}=3/rev$ and (**c**) $f_{fault}=1/rev$, respectively. The points corresponding to changes in contact pressure are indicated in (**a**). The test status is colour coded, as shown in the legend. The black vertical lines indicate the moments of PAUT inspections. The break points highlighting decisions, *D*_1,_ are marked by red crosses in (**b**,**c**).

**Figure 8:**
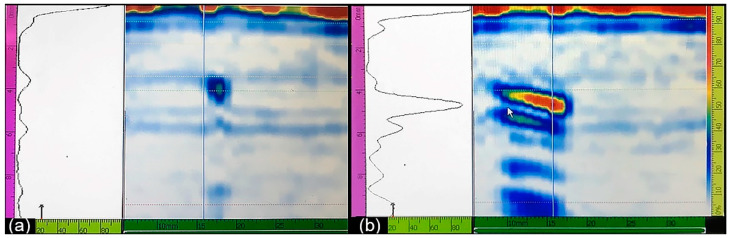
Screenshots from the Olympus OMNISCAN MX2 ultrasonic control box, using the Dual Linear Array 7.50L32-REX1-IHC probe, showing (**a**) the initial crack of approximately 0.5 mm length and (**b**) the same crack at the final stage with the transverse length of 10 mm.

The most notable findings from the durability test are represented in Figure 7. The colour code is shown in the legend: green denotes the “healthy” state of the roller when no defects were detected by any means; yellow segments indicate the “idle” status when the testing rig was paused for maintenance and adjustments; pink segments with different densities indicate different “damage” stages corresponding to the progressive subsurface crack propagation according to periodic ultrasonic inspections; and vertical black lines indicate the moments when the test was interrupted for visual and PAUT examinations of the test roller. The PAUT investigations revealed the first subsurface crack (SSC) of approximately 0.5 mm at 2.8 × 10^7^ fatigue cycles, as is shown in Figure 8a. After nucleation, the crack kept growing steadily to approximately 5 mm in length (Figure 8b) before the test was terminated and the roller was cut for metallographic examination and verification of subsurface cracking, as will be discussed in the next section.

Figure 7a shows the behaviour of the conventional AE rms value, which apparently leaves no hope of finding any signature of early damage in a realistically noisy environment, at least as long as the damage initiates and propagates in the subsurface layer before becoming critical at the surface. The proposed algorithm, however, successfully captured the initiation and propagation of the subsurface cracks. The following parameters were set for the detector: $l_{w}$ = 1000, $o_{w}$ = 500, c =10, and $l_{B}$ = 300.

The first positive detector decision,$\;D_{1}$ (*defect detected)*, is indicated in Figure 7b by the red cross. Beyond this breakpoint, the same decisions are consistently obtained until the end of the test. The corresponding PIS verification of the detector decision is shown above in Figure 6. Apparently, it signifies the faulting process that occurs with the frequency $f_{fault}=3/rev$, which is plausibly expected for the damage related to the test roller, as the only place in the test machine that can cause this behaviour is a point on the test specimen perimeter passing the support rollers. During the test, no damage was detected on the surface of the roller. As the subsurface cracks were reliably detected by PAUT inspections, we conclude that the proposed method captured the subsurface cracking with high confidence.

**Figure 9:**
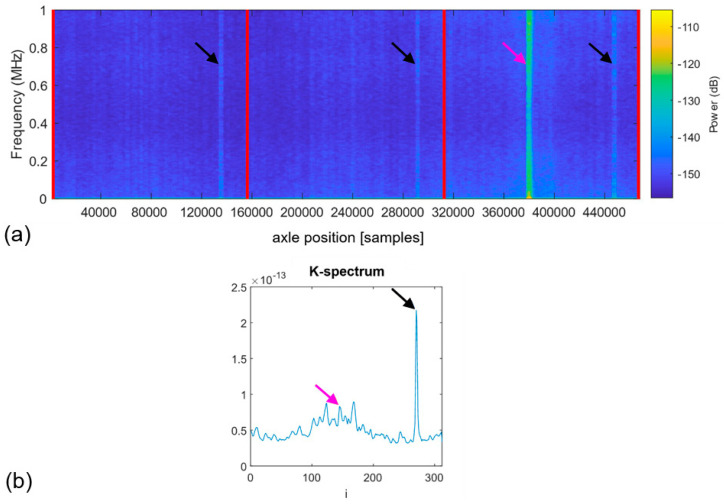
Typical Pulse Integrated Spectrogram corresponding to one axle revolution divided into three segments of equal length and marked by vertical red lines (**a**) and the *K*-spectrum corresponding to the middle segment (**b**) observed during the late stage of the test, at 52.8 × 10^6^ fatigue cycles: two independent damaging processes evolve in parallel. The black arrows indicate the spectral lines corresponding to $f_{fault}=3/rev$ and the pink arrows highlight the faults appearing at $f_{fault}=1/rev$.

It should be noted that Figure 7c makes it evident that, besides the subsurface damage propagating in the test roller, another source of hidden periodic signals does exist with the characteristic frequency of 1 pulse per revolution, i.e., with $f_{fault}=1/rev$. This source is apparently not associated with the behaviour of the test roller. The independence of this kind of damage process from the one featured by the frequency $f_{fault}=3/rev$ is corroborated by the fact that the second process commences later in time (compare Figure 7b,c), and evolves differently, as can be seen by trends in the *L* vs. cycles plots. The pulse integrated spectrogram in Figure 9 corresponds to the later stage of the test beyond the breakpoint,$\;D_{1}$ (*defect detected)*, in Figure 7c. In addition to the three familiar spectral lines seen earlier in Figure 6, a pronounced spectral line corresponding to the periodic AE behaviour with $f_{fault}=1/rev$ can appear on the spectrograms such as those shown in Figure 4 and Figure 9 (marked with pink arrows). This line is notably more difficult to notice in the *K*-spectrum, and it is only visible within the broad peak marked by the pink arrow. As this particular detector seeks a defect with $f_{fault}=3/rev$, this behaviour illustrates the importance of outlier removal to achieve a better resolution of the sought frequencies.

As a matter of discussion, Figure 7a shows that the rms value of the unfiltered signal does not correlate with either of the two types of faults found in AE waveforms by the proposed detector. Therefore, rms or similar features are not good candidates for training any classifier, including those based on machine learning algorithms. Furthermore, the fault behaviour, which is characterised by $f_{fault}=1/rev$, has a maximum *L*-value that is approximately 17 times greater than the maximum of *L* in the sought behaviour with $f_{fault}=3/rev$. This means that the cause of this behaviour is likely not associated with the subsurface or surface faults in the test roller. These pulses are notably different from those with $f_{fault}=3/rev$; they are significantly stronger by far. There are several locations on the testing rig that might cause pulses of this kind, including the gearbox, motor, shaft couplers, etc. However, the origin of this behaviour is not yet precisely established. Another corollary is that even though there are three times more pulses integrated to produce the *L* vs. cycles plot for $f_{fault}=3$, compared to that for $f_{fault}=1/rev$, AE pulses from subsurface cracks are so weak that detector decisions must be verified (with PIS or similar tools) to ensure that what is detected is the sought damage-induced behaviour. The random noisy pattern generated at the AE sensor output during the operation of a multi-component rotating machine is affected by many factors. Thus, it can hardly be explained rationally, giving rise to multiple possible interpretations and/or misinterpretations. For subsurface crack detection, one cannot trust non-destructive testing platforms or health indicators if they are entirely data-driven and lacking a proper verification tool (such as the PIS proposed here), which is needed to unveil the deterministic knowledge-based information hidden in noisy data. This is why the significance of a verification method in the data processing chain cannot be overvalued.

### 4.2. Metallographic Examination and Confirmation of Subsurface Cracking

**Figure 10:**
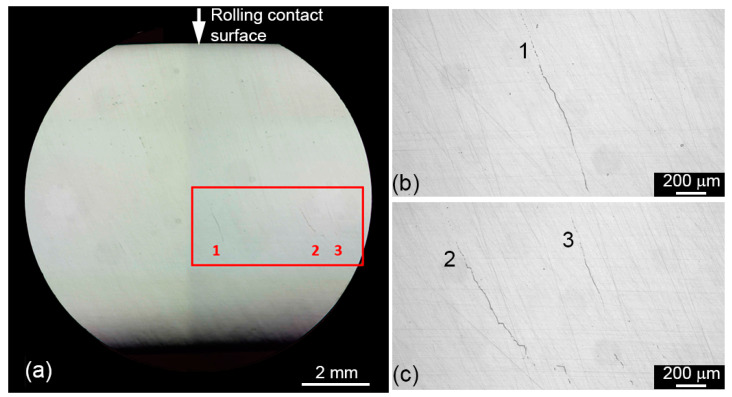
(**a**) Subsurface cracks revealed in metallographic sections of the test roller and (**b**,**c**) magnified views of three subsurface roller-fatigue induced cracks marked 1–3.

Post-mortem sectioning of the test roller revealed that three parallel cracks developed close to each other during the durability test. They were identified as one crack by the regular PAUT inspections. The cracks’ location is approximately 4 mm below the contact surface, and the distance between the outermost edges of the three cracks is approximately 6 mm in the rolling direction (see Figure 10, for example). The transverse extension of the cracks, which is believed to be larger compared to the rolling direction, was not examined using the destructive slicing procedure. The first subsurface crack identified by PAUT was discovered after 1.1 × 10^6^ fatigue cycles at 2000 MPa. Recall that 2.7 × 10^7^ fatigue cycles were accumulated at 1800–1900 MPa prior to testing at 2000 MPa. The initial crack width of the first observed subsurface crack was estimated to be 0.5 mm. After the durability test was finished, no surface spalling, pitting, or change in surface roughness was identified on the test specimen contact surface by regular visual inspections.

## 5. Final Remarks, Conclusions, and Future Scopes

A durability test was conducted to successfully induce subsurface cracks caused by rolling contact fatigue in a case-hardened test roller. During testing, subsurface crack initiation and propagation were systematically monitored using phased array ultrasonic testing. After the test was completed, the test specimen was sliced perpendicularly to the axis and microscopically inspected. Several subsurface cracks were found in coherence with ultrasonic examinations, whereas no surface damage was observed.

AE waveforms of 2 s duration were periodically recorded during the entire fatigue test. The signal processing scheme proposed here is fundamentally founded on the knowledge available regarding the rotating mechanical system under consideration. A specific data exploration algorithm informed by the precise rotation speed and/or axle position was utilised to extract and verify periodic patterns from AE data. A detector relying on the periodic integration of specifically located AE pulses was developed to detect the appearance of subsurface cracks at an early stage and follow crack growth behaviour.

The effectiveness of the proposed detector is evident from the simple juxtaposition of detector decisions with the results of ultrasonic investigations. To avoid false alarms and to cross-validate the positive detector’s decisions, the Pulse Integrated Spectrogram verification tool was proposed and used to check whether the detected behaviour originated from the sought defect or not. It was demonstrated that subsurface cracks can be reliably detected in rotating components at the early stage of their evolution, before they reach the surface and cause serious damage.

Nonetheless, we must reiterate that the high degree of confidence achieved in the present work relies on the specifically designed data exploration tools using prior knowledge about the particular rotating machine tested. In this sense, the presented technique differs from a wealth of entirely data-driven approaches including those based on machine learning principles. However, this does not substantially reduce the applicability of our “data-inspired” methodology, since the data required for its successful implementation refer simply to the specific geometry and rotation speed of the rotating components—information that is usually readily available to designers, engineers, and researchers. Nonetheless, reliance on the temporal repeatability of damage-related AE events is admittedly the chief limitation of our method in its present form. In other words, the entire approach depends heavily on the rotation stability of all rollers. Therefore, the logical continuation of this work will be to adapt the proposed detector to a rotating machine with a dynamically variable rotation speed measured concurrently with AE waveforms. Detector parameters such as the window length, shape, and overlap can be optimised further; the strategy for this optimisation has yet to be developed. Another limitation of the present laboratory work is that the AE sensor was located in close proximity to the test specimen, which is not always feasible in industrial settings. Thus, for practical implementation of the proposed approach, the challenges associated with AE transmission through specific complex industrial structures need to be overcome [82].

Despite the concerns raised in the introduction, machine learning or deep learning is not completely negated, and despite the demonstrated success story, the present knowledge-inspired approach is not completely supported; their competitiveness and mutual complementariness can be combined in the future. The synergy from such a combination is deemed to arise owing to the high confidence provided by the proposed knowledge-based approach paired with the versatility and adaptability of deep learning. As a preliminary notice, we can say that in our ongoing research we explore the capacity of alternative data-driven methods based on artificial intelligence and deep learning strategies applied to the same dataset. The comparative results will be presented elsewhere.

## Figures and Tables

**Table 1:** Interpretation of the window function.

		Recorded Axle Revolutions			
		Sub Window 1	Sub Window 2	Sub Window 3	⋯
Axle position	Window 1	Pos1, Rev1	Pos1, Rev2	Pos1, Rev3	⋯
	Window 2	Pos2, Rev1	Pos2, Rev2	Pos2, Rev3	⋯
	Window 3	Pos3, Rev1	Pos3, Rev2	Pos3, Rev3	⋯
	⋮	⋮	⋮	⋮	⋱

## Data Availability

The datasets generated and analyzed during this study are not currently available to the public due to the corporate policies of industrial project partners.
